# Syntheses and structures of two coordination polymers formed by Ni(cyclam)^2+^ cations and sulfate anions

**DOI:** 10.1107/S2056989024012337

**Published:** 2025-01-03

**Authors:** Liudmyla V. Tsymbal, Irina L. Andriichuk, Lucian G. Bahrin, Yaroslaw D. Lampeka

**Affiliations:** a L. V. Pisarzhevskii Institute of Physical Chemistry of the National Academy of Sciences of Ukraine, Prospekt Nauki 31, 03028, Kyiv, Ukraine; b "Petru Poni" Institute of Macromolecular Chemistry, Aleea Gr. Ghica Voda 41A, RO 700487 Iasi, Romania; University of Aberdeen, United Kingdom

**Keywords:** crystal structure, coordination polymer, cyclam, nickel, sulfate, hydrogen bonds

## Abstract

The asymmetric units of *catena*-poly[[(1,4,8,11-tetra­aza­cyclo­tetra­decane-κ^4^*N*^1^,*N*^4^,*N*^8^,*N*^11^)nickel(II)]-μ_2_-sulfato-κ^2^*O*^3^:*O*^4^], [Ni(SO_4_)(C_10_H_24_N_4_)]_*n*_ (**I**), and *catena*-poly[[(1,4,8,11-tetra­aza­cyclo­tetra­decane-κ^4^*N*^1^,*N*^4^,*N*^8^,*N*^11^)nickel(II)]-μ_2_-sul­fato-κ^2^*O*^3^:*O*^4^] hemi[4,4′,4′′,4′′′-(2,2′,4,4′,6,6′-hexa­methyl-[1,1′-biphen­yl]-3,3′,5,5′-tetra­yl)tetra­benzoic acid] nona­hydrate], {[Ni(SO_4_)(C_10_H_24_N_4_)]_2_·C_46_H_38_O_8_·18H_2_O}_*n*_ (**II**), consist of two crystallographically unique centrosymmetric macrocyclic dications and a sulfate dianion. In **II** it includes additionally a mol­ecule of the undissociated acid (2,2′,4,4′,6,6′-hexa­meth­yl[1,1′-biphen­yl]-3,3′,5,5′-tetra­yl)tetra­(benzoic acid) located on a crystallographic twofold axis and nine highly disordered water mol­ecules of crystallization. In both compounds, the metal ions are coordinated in the equatorial plane by the four secondary N atoms of the macrocyclic ligand, which adopts the most energetic­ally stable *trans*-III conformation. Two O atoms of the sulfate anions occupy the *trans*-axial positions resulting in a slightly tetra­gonally distorted *trans*-NiN_4_O_2_ octa­hedral coordination geometry. The crystals of both compounds are composed of parallel coordination polymeric chains running along the [101] and [100] directions in **I** and **II**, respectively. The distances between the neighboring metal ions in the chains are significantly different [6.5121 (6) Å in **I** and 6.0649 (3) Å in **II**] and this peculiarity is explained by the different spatial directivity of the Ni—O coordination bonds (different S—O—Ni angles). As a result of the C—H⋯O hydrogen bonds between the methyl­ene groups of the macrocyclic ligands and the non-coordinated O atoms of the sulfate anion, the coordination-polymeric chains in **I** are arranged in the two-dimensional layers oriented parallel to the (010) and (101) planes, the inter­section of which provides the three-dimensional coherence of the crystals. The three-dimensional supra­molecular structure of the crystals **II** is determined by the network of strong hydrogen bonds formed by the carb­oxy­lic acid and the non-coordinated O atoms of the sulfate anions.

## Abstract 2

The coordination polyhedra of the complex cations in the one-dimensional title coordination polymers represent tetra­gonally distorted *trans*-NiN_4_O_2_ octa­hedra with the four N atoms of the macrocyclic ligand forming the equatorial plane and two O atoms of the sulfate anions occupying the axial positions.

## 1. Chemical context

Nickel(II) complexes of 14-membered tetra­dentate aza­macrocyclic ligands, in particular, cyclam and its analogues (cyclam = 1,4,8,11-tetra­aza­cyclo­tetra­decane, C_10_H_24_N_4_, *L*), are widely used in the formation of coordination polymers and metal–organic frameworks based on oligo­carboxyl­ate linkers, which possess many promising applications (Lampeka & Tsymbal, 2004 ▸; Suh & Moon, 2007 ▸; Stackhouse & Ma, 2018 ▸). At the same time, examples of coordination polymers formed by these Ni^II^-containing nodes and simple inorganic oxoanions are rare and are limited mainly to compounds containing bridging chromate ligands (see *Database survey*). Surprisingly, no polymers formed by the sulfate dianion and tetra­aza­macrocyclic Ni^II^ cations have been described to date. At the same time, it can be expected that the formation of structures containing two different types of bridging ligand (*i.e.*, organic carboxyl­ates and inorganic oxoanions) will enrich the topological variability of the coordination polymers and their functional characteristics. To check such a possibility, we conducted the reaction between an excess (8:1) of [Ni(*L*)](ClO_4_)_2_ and (2,2′,4,4′,6,6′-hexa­meth­yl[1,1′-biphen­yl]-3,3′,5,5′-tetra­yl)tetra­(benzoic acid) (H_4_A) in the presence of Na_2_SO_4_.
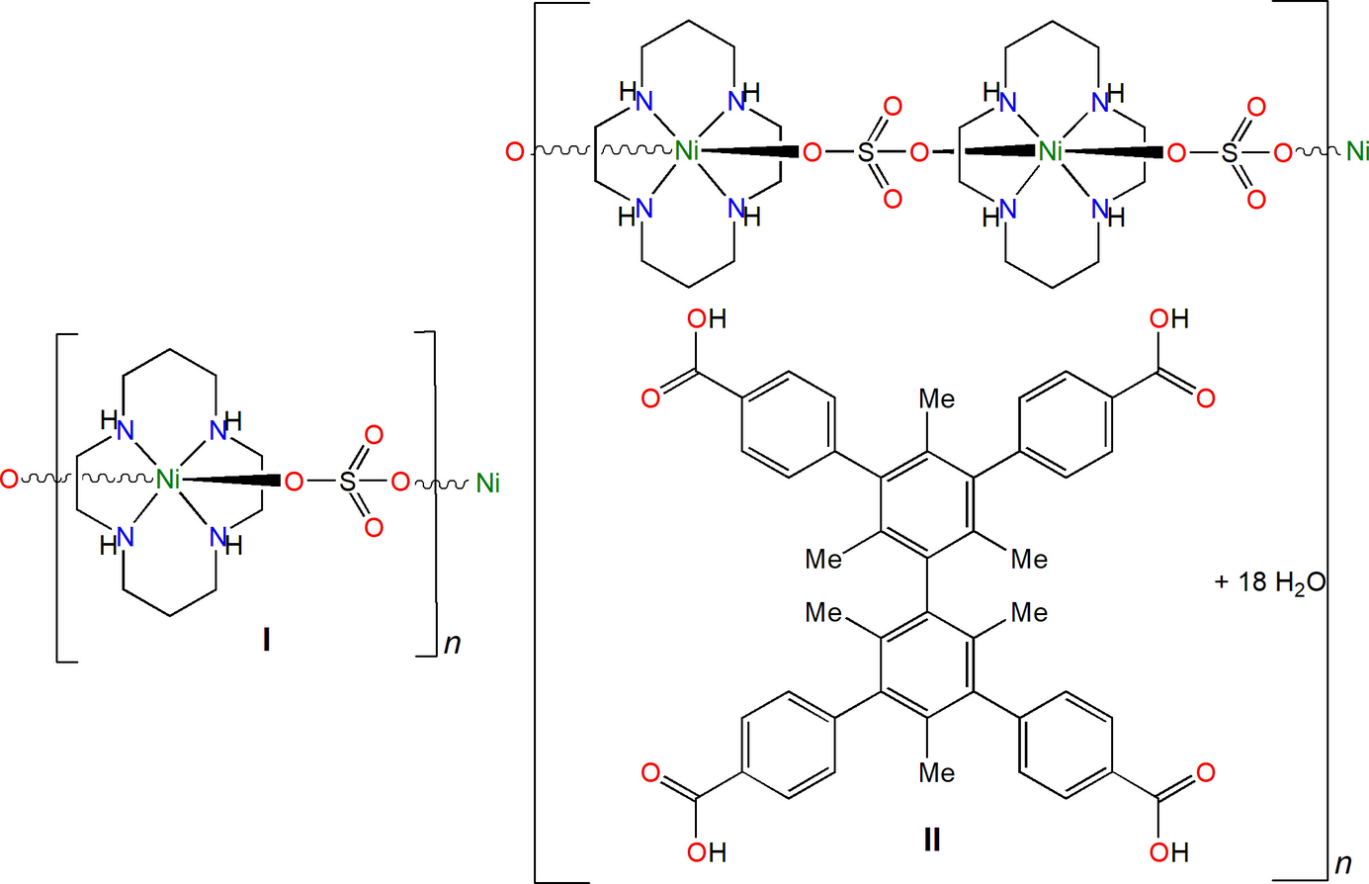


The present work describes the preparation and structural characterization of the products of this reaction which are the first representatives of polymeric complexes formed by the [Ni(*L*)]^2+^ cation and SO_4_^2–^ anions, namely, *catena*-poly[[(1,4,8,11-tetra­aza­cyclo­tetra­decane-κ^4^*N*^1^,*N*^4^,*N*^8^,*N*^11^)nickel(II)]-μ_2_-sulfato-κ^2^*O*^3^:*O*^4^], [Ni(SO_4_)(C_10_H_24_N_4_)]_*n*_ (**I**), and *catena*-poly[[(1,4,8,11-tetra­aza­cyclo­tetra­decane-κ^4^*N*^1^,*N*^4^,*N*^8^,*N*^11^)nickel(II)]-μ_2_-sul­fato-κ^2^*O*^3^:*O*^4^] hemi[4,4′,4′′,4′′′-(2,2′,4,4′,6,6′-hexa­methyl-[1,1′-biphen­yl]-3,3′,5,5′-tetra­yl)tetra­benz­oic acid] nona­hydrate], {[Ni(SO_4_)(C_10_H_24_N_4_)]_2_·C_46_H_38_O_8_·18H_2_O}_*n*_ (**II**).

## 2. Structural commentary

**Figure 1:**
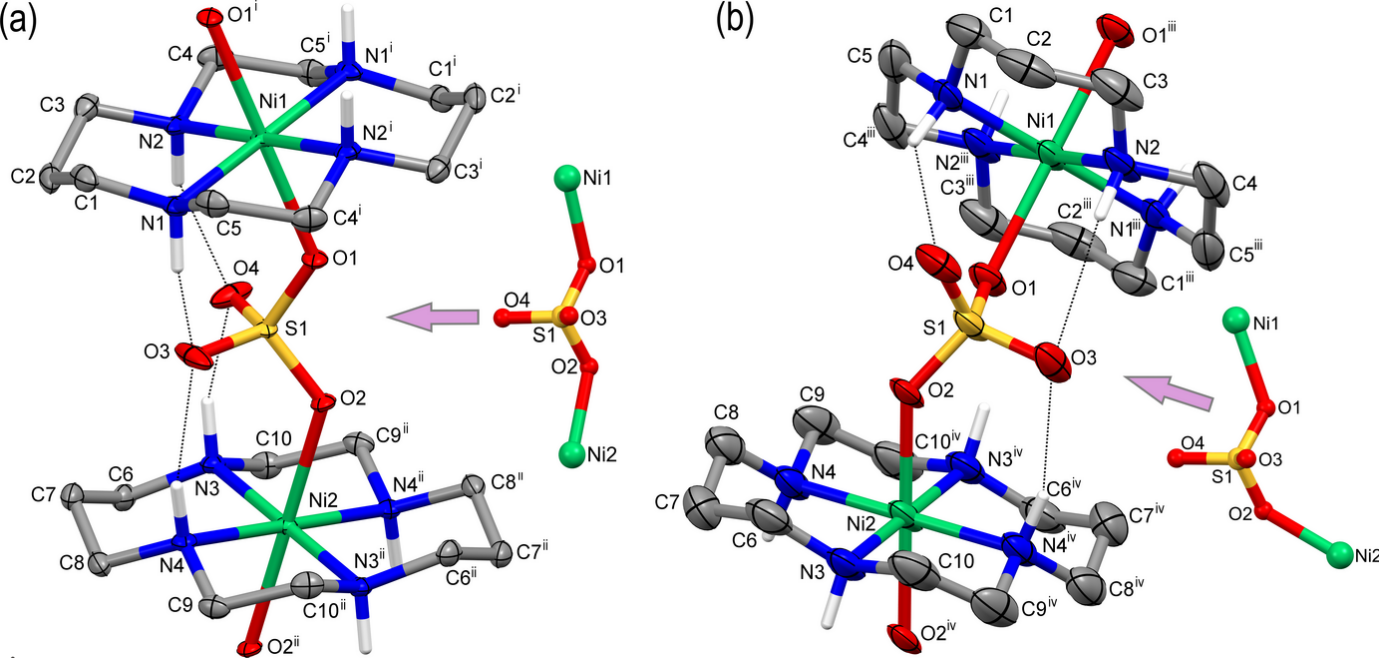
The extended asymmetric units involving complex cations in (*a*) **I** and (*b*) **II** showing the atom-labeling scheme (displacement ellipsoids are drawn at the 30% probability level, C-bound H atoms are omitted for clarity, intra­molecular hydrogen bonds are shown as dotted lines). The relative orientations of the coordination links Ni1—O1—SO_2_—O2—Ni2 in each compound are shown on the right. Symmetry codes: (i) −*x*, −*y* + 1, −*z* + 1; (ii) −*x* + 1, −*y* + 1, −*z* + 2; (iii) −*x* + 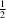, −*y* + 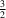, −*z* + 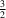; (iv) −*x* + 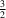, −*y* + 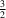, −*z* + 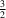.

The asymmetric units of both compounds contain two crystallographically unique centrosymmetric macrocyclic cations [Ni(*L*)]^2+^ and one sulfate anion (Fig. 1 ▸). In **II** it includes additionally the mol­ecule of the acid H_4_A and, according to SQUEEZE calculations, nine highly disordered water mol­ecules of crystallization.

The coordination environments of the metal ions in **I** and **II** are very similar. The Ni^II^ ions are equatorially coordinated to the four secondary N atoms of the macrocycle *L*, while the axial positions in the coordination spheres are occupied by the O atoms of the sulfate anions. Since the Ni—N bond lengths, which are typical of high-spin Ni^II^ 3*d*^8^ electronic configuration, are slightly shorter than the Ni—O ones (Table 1 ▸), the coordination polyhedra in both compounds can be described as tetra­gonally elongated *trans*-NiN_4_O_2_ octa­hedra. Inter­estingly, the Ni—O distances are nearly equal in **I**, while they differ significantly in **II** (Table 1 ▸).

The macrocyclic ligands *L* adopt the most energetically stable *trans*-III (*R,R,S,S*) conformation (Barefield *et al.*, 1986 ▸) with the five-membered (N—Ni—N bite angles *ca* 85°) and six-membered (N—Ni—N bite angles *ca* 95°) chelate rings being in *gauche* and *chair* conformations, respectively (Table 1 ▸).

The NiN_4_ coordination moieties in **I** and **II** are strictly planar because of the location of the metal ions on crystallographic inversion centers. The axial Ni—O bonds are nearly orthogonal to the NiN_4_ planes (deviations of the angles N—Ni—O from 90° do not exceed 4°). Analogously to other complexes of the Ni^II^ macrocyclic cations and carboxyl­ate ligands (Tsymbal *et al.*, 2021 ▸) the Ni—O coordination inter­actions in **I** and **II** are reinforced by intra­molecular hydrogen bonds between the secondary NH atoms of the amine groups and the non-coordinated O atoms of the sulfate anions (Fig. 1 ▸, Tables 2 ▸ and 3 ▸).

In both complexes, the sulfate ligands display a μ_2_-bis-monodentate bridging mode resulting in the formation of linear (*i.e*., an angle Ni⋯Ni⋯Ni of 180°), parallel, coordination-polymeric chains running along the [101] and [100] directions in **I** and **II**, respectively. Despite close similarities in coordination bond lengths in both compounds, there are several differences in the structures of the polymeric chains connected with the mutual orientation of the constituents. In particular, the angle between the mean NiN_4_ planes of the structurally non-equivalent macrocyclic cations in **I** is 31.44 (9)°, while in **II** it is 41.6 (2)°. Additionally, the angles between the long axes of these macrocyclic cations passing through the symmetry-related central C atoms of the tri­methyl­ene fragments (C2 or C7) and the Ni^II^ ion are 7.51 (9) and 56.2 (2)° in **I** and **II**, respectively. Besides, the distances between the neighboring metal ions in the chains are significantly different [6.5121 (6) Å in **I** and 6.0649 (3) Å in **II**]. Obviously, this feature is explained by different mutual spatial directivity of the Ni—O coordination bonds. That is to say, though the angles S1—O1—Ni1 are nearly equal in **I** and **II** [126.17 (11) and 127.61 (19)°, respectively], the angles S1—O2—Ni2 differ significantly [126.36 (11) and 135.8 (2)°] (Fig. 1 ▸).

**Figure 2:**
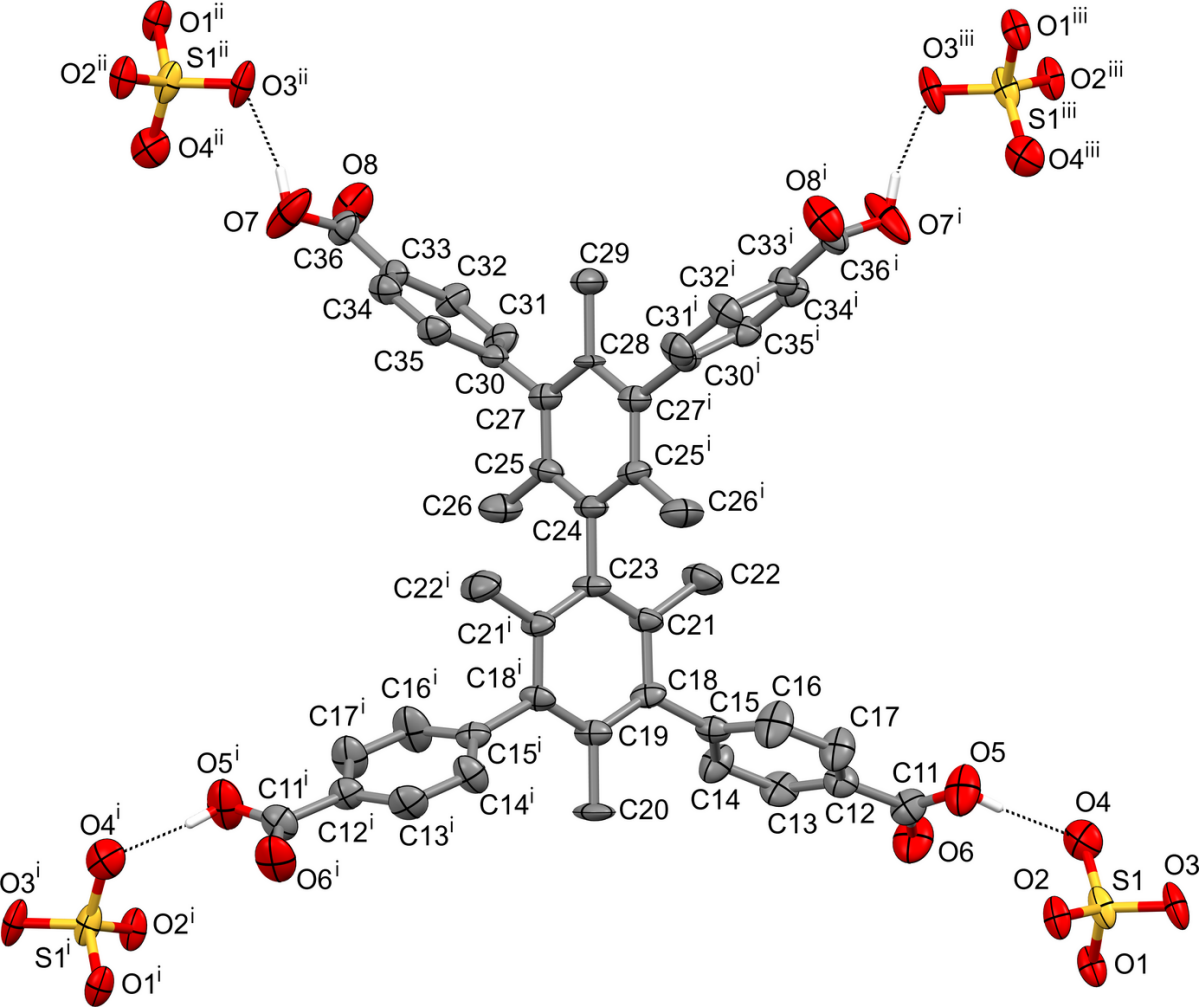
The conformation of the acid H_4_A in **II** with the hydrogen bonds (dotted lines) it forms with the sulfate anions (displacement ellipsoids are drawn at the 30% probability level, C-bound H atoms are omitted for clarity). Symmetry codes: (i) −*x* + 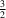, *y*, −*z* + 1; (ii) *x*, −*y* + 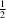, *z* − 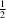; (iii) −*x* + 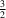, −*y* + 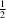, −*z* + 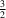.

The clathrated mol­ecule of the acid H_4_A in the crystal of **II** is localized on a crystallographic twofold axis passing through the C20/C29 carbon atoms (Fig. 2 ▸) and is characterized by the non-planar structure manifesting itself in substantial mutual tilting of the aromatic rings. This is caused by repulsive inter­actions of the hydrogen atoms of the methyl substituents between themselves [the angle between the mean planes of the central tri­methyl­benzene fragments is 75.3 (2)°] or with the hydrogen atoms of the pendant aromatic rings [the angles between the mean planes of tri­methyl­benzene rings and the lateral carboxyl-substituted ones are 71.4 (2) and 77.8 (2)°]. The latter values are close to those observed in the complex of a structurally related tri­phenyl­phospho­nic acid built on a tri­methyl­benzene core (Tsymbal *et al.*, 2022 ▸). The angles C11—*Cg*—C11(−*x* + 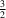, *y*, −*z* + 1) and C36—*Cg*—C36(−*x* + 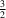, *y*, −*z* + 1) are 131.2 (1) and 114.8 (1)° (*Cg* represents the centroid of the corresponding tri­methyl­benzene ring) and, because of the tilting of these rings, the mol­ecule H_4_A as a whole possesses a tetra­hedron-like shape. The carb­oxy­lic acid groups in H_4_A are close to coplanar with their corresponding benzene rings (the angles between their mean planes are smaller than 8°) and are non-delocalized as indicated by the large differences in the lengths of the C—OH (*ca* 1.30 Å) and C=O (*ca* 1.20 Å) bonds.

## 3. Supra­molecular features

**Figure 3:**
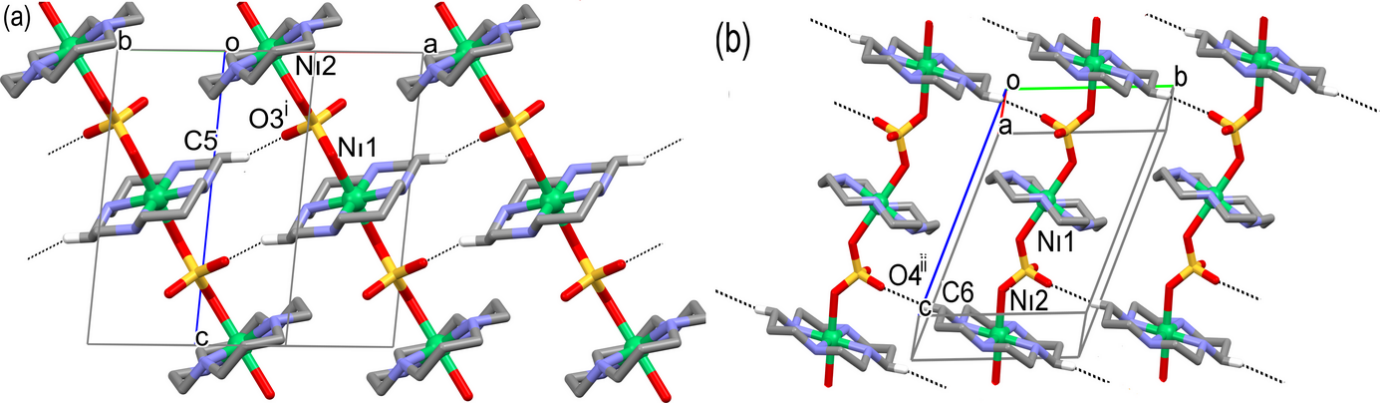
The hydrogen-bonded (dashed lines) sheets in **I** parallel to the (*a*) (010) and (*b*) (10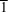) planes (only atoms H5*A* and H6*B* participating in inter­chain hydrogen bonding are shown). Symmetry codes: (i) −*x* + 1, −*y* + 1, −*z* + 1; (ii) −*x* + 1, −*y* + 2, −*z* + 2.

The three-dimensional coherence of the crystal of **I** is supported by weak C—H⋯O hydrogen bonds between the C5 and C6 methyl­ene groups belonging to the structurally non-equivalent macrocyclic cations and the non-coordinated O3 and O4 atoms of the sulfate anion (Table 2 ▸). In particular, the polymeric chains in **I** are arranged in pseudo layers oriented parallel to the (010) plane due to C5—H5*A*⋯O3 contacts (Fig. 3 ▸*a*). Simultaneously, similar layers, though oriented parallel to the (10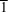) plane (Fig. 3 ▸*b*), are formed as a result of the C6—H6*B*⋯O4 inter­actions. The inter­section of these layers results in the formation of a three-dimensional system of hydrogen bonds in the crystal. The shortest distance between the Ni^II^ ions in neighboring chains is *ca* 8.0 and 8.3 Å in the former and the latter cases, respectively. It is noteworthy that both the non-coordinated O atoms of the sulfate anion in **I** are saturated by hydrogen bonds, acting as triple proton acceptors. According to *PLATON* calculations (Spek, 2020 ▸), the crystals of **I** are non-porous.

**Figure 4:**
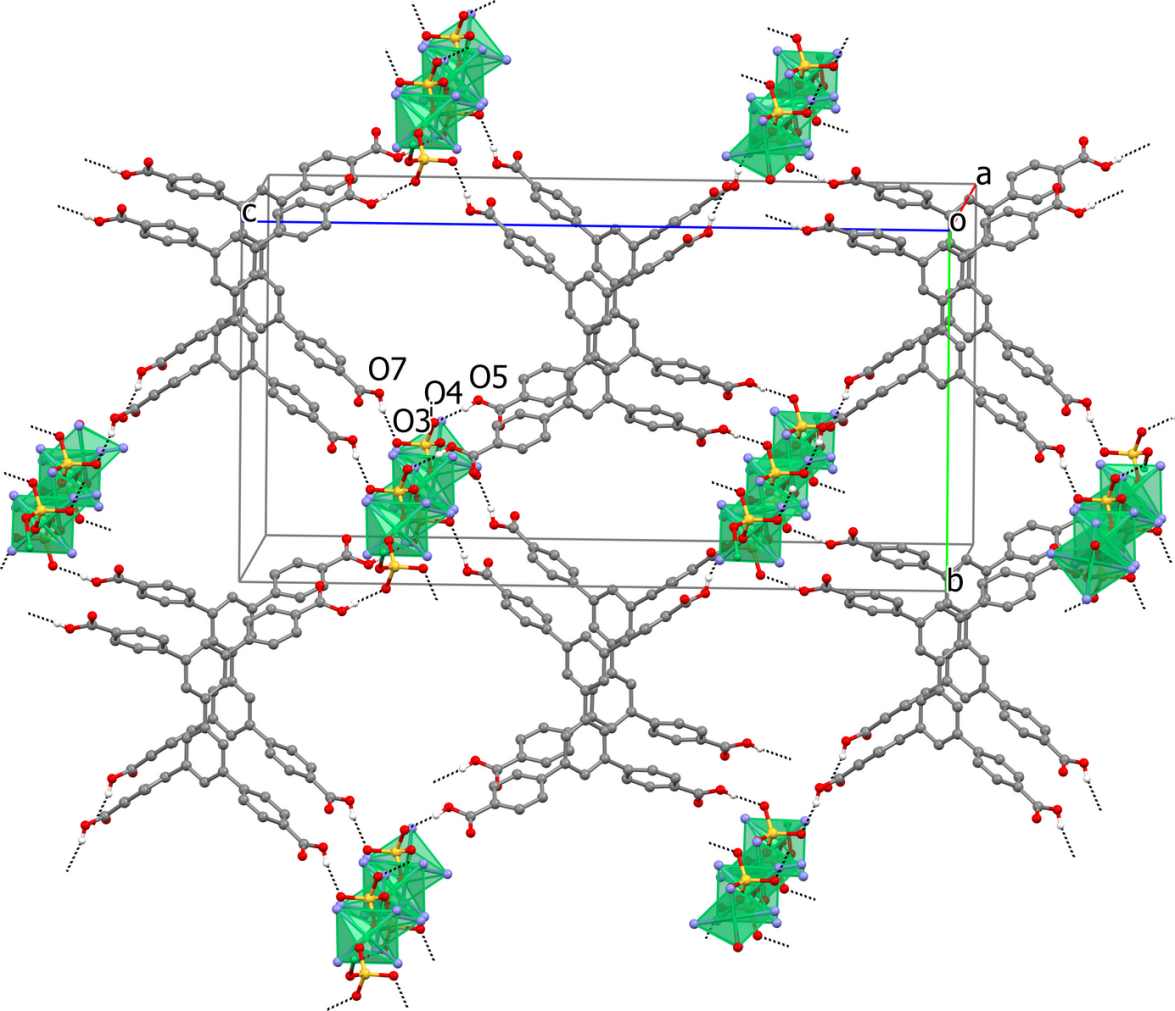
Fragment of the crystal structure of **II** showing the hydrogen bonds (dashed lines) between carboxyl­ate groups of the acid H_4_L and the non-coordinated oxygen atoms of the sulfate anions. H atoms at C atoms and methyl­ene groups in benzene rings are not shown. The coordination environment of the Ni^II^ ions is shown in polyhedral presentation. Symmetry code: (i) *x*, −*y* + 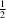, *z* − 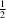.

A pivotal role in the formation of the extended structure of the crystals of **II** is played by the carb­oxy­lic acid H_4_A. Acting as the proton donor, it forms strong hydrogen bonds with the non-coordinated O3 and O4 atoms of the sulfate anion (Table 3 ▸), which belong to four different polymeric chains. These chains act as pillars and, in turn, the anions of each asymmetric units inter­act with four mol­ecules of the acid (Fig. 4 ▸). At the same time, the tetra­hedral shape of this mol­ecule prevents the formation of any two-dimensional aggregates, thus resulting in a three-dimensional system of hydrogen bonds in the crystals **II**. As estimated by *PLATON* (Spek, 2020 ▸), the volume of the solvent-accessible void in **II** in the form of isolated cavities equals 1667 Å^3^ (20.9% of the unit-cell volume) which, according to SQUEEZE calculations, are filled with eighteen highly disordered water mol­ecules of crystallization.

## 4. Database survey

Data concerning the crystal structure of sulfate complexes of the Ni(*L*) cation(s) are very limited. In particular, the Cambridge Structural Database (CSD, Version 5.45, last update September 2024; Groom *et al.*, 2016 ▸) contains characterization of the only one non-polymeric Ni^II^ complex anion *trans*-[Ni^II^(*L*)(SO_4_)_2_]^2−^ (refcode FAFLUV; Churchard *et al.*, 2010 ▸) and two compounds of the Ni^III^ complex cation *trans*-[Ni^III^(*L*)(HSO_4_)_2_]^+^ (RIGFUM and RIGGIB; Morrison *et al.*, 2023 ▸). Additionally, the one-dimensional coordination polymer based on the *trans*-[Ni^III^(*L*)(SO_4_)_2_]^−^ unit has also been described (RIGGEX; Morrison *et al.*, 2023 ▸). It is noteworthy that, despite the different chemical nature of FAFLUV and **I** and **II** (*i.e.* non-polymeric and polymeric, respectively), the coordination bond lengths in all complexes are very similar (*cf*. average Ni—N and Ni—O distances in FAFLUV of 2.07 and 2.15 Å, respectively, with the corresponding parameters presented in Table 1 ▸).

Despite the lack of structurally characterized polymeric Ni^II^(*L*)–sulfate compounds, there is one example of a polymeric complex of this cation with the chromate anion – a ligand that is closely related to sulfate (NAYWUF; Oshio *et al.*, 1997 ▸). In addition, a number of polymeric complexes of the [Ni(di­aza­cyclam)]^2+^ macrocyclic cation [di­aza­cyclam = (3,10)-*R*_2_-1,3,5,8,10,12-hexa­aza­cyclo­tetra­deca­ne] with the CrO_4_^2–^ anion have been described [GUJNUU; Kim *et al.*, 2000 ▸, and GUJNUU01, Gu *et al.*, 2008 ▸ (*R* = 2-hy­droxy­eth­yl); RAHZAD; Ou *et al.*, 2011 ▸ (*R* = prop­yl); VEWWEB and VEWWIF; Shin *et al.*, 2013 ▸ (*R* = *S,S*- or *R*,*R*-1-phenyl­eth­yl)], as well as the coordination polymer with the molybdate anion [GUJPAC; Kim *et al.*, 2000 ▸ (*R* = 2-hy­droxy­eth­yl)].

In general, the crystal structures of all of the above-mentioned polymeric complexes of the Ni^II^ ion are rather similar and related to **I** and **II**. Their crystals are also built from parallel polymeric chains and the lengths of the Ni^II^—O axial coordination bonds (2.06–2.10 Å) are only slightly shorter than those observed in **I** and **II**. This feature, together with strictly linear (NAYWUF and RAHZAD) or close to linear (other complexes) arrangement of the Ni^2+^ ions in the chains and a similar mode of coordination of the MO_4_^2–^ anions to that observed in **I**, results in a narrow spread of the intra­chain metal–metal distances (6.6–6.8 Å). Inter­estingly, though the average Ni^III^—O bond length in RIGGEX (2.11 Å) does not differs significantly from that observed in **I** or **II**, a shorter Ni⋯Ni intra­chain distance (6.28 Å) in the former polymer is explained by the essential non-linearity of the chains (the angle Ni⋯Ni⋯Ni is *ca* 164°).

The acid H_4_A has been used for the preparation of several polymeric compounds, including complexes of Zr^IV^ and Hf^IV^ (Yan *et al.*, 2018 ▸; Lv *et al.*, 2019 ▸; Zhang *et al.*, 2020 ▸), Eu^III^ (Lv *et al.*, 2021 ▸), Cd^II^ (Wang *et al.*, 2019 ▸) and alkali- and alkaline-earth metal ions (Bahrin *et al.*, 2019 ▸; Li *et al.*, 2022 ▸). Additionally, the structures of the uncoordinated acid in solvated (RAXXIY; Moorthy *et al.*, 2005 ▸) and unsolvated (HEGCEF; Wang *et al.*, 2021 ▸) states as well as in mono- and dianionic forms (BOVNEI; Bahrin *et al.*, 2019 ▸) have been reported. The comparison of structural data available in the literature for uncoordinated H_*n*_A^(4–*n*)–^ with those of H_4_A in **II** demonstrates rather minor differences in inter­atomic distances and angles and in general shapes of the ions and mol­ecules, which obviously is connected with their low conformational flexibility caused by strong intra­molecular inter­atomic repulsions.

## 5. Synthesis and crystallization

All chemicals and solvents used in this work were purchased from Sigma–Aldrich and used without further purification. The acid H_4_A was synthesized according to a procedure described previously (Bahrin *et al.*, 2019 ▸). The complex [Ni(*L*)](ClO_4_)_2_ was prepared from ethanol solutions as described in the literature (Bosnich *et al.*, 1965 ▸).

The coordination polymers **I** and **II** were prepared as by-products of the reaction between the excess of the perchlorate salt of [Ni(*L*)]^2+^ cation and the acid H_4_A (8:1) in the presence of Na_2_SO_4_ as follows.

A solution of H_4_A (35 mg, 0.050 mmol) in 5 ml of DMF was mixed with a solution of [Ni(*L*)](ClO_4_)_2_ (183 mg, 0.40 mmol) dissolved in 5 ml of a DMF/H_2_O mixture (1:1 by volume). Na_2_SO_4_ (100 mg, 0.70 mmol) was then added and a solution was heated at 353 K for 30 min and left to stand at ambient conditions. Light-violet prisms of **I**, which formed in a week, were filtered off, washed with small amounts of methanol and diethyl ether, and dried in air. Yield: 14 mg (10% based on nickel complex). Analysis calculated for C_10_H_24_N_4_NiO_4_S: C 33.83, H 6.81, N 15.58%. Found: C 33.71, H 6.92, N 15.39%.

Refrigerating the mother liquor obtained after filtering off complex **I** resulted in the formation of **II** after one day in the form of nearly colorless light-pink plates. These were filtered off, washed with small amounts of methanol and diethyl ether, and dried in air. Yield: 22 mg (7% based on nickel complex). Analysis calculated for C_66_H_122_N_8_Ni_2_O_34_S_2_: C 45.22, H 7.01, N 6.39%. Found: C 45.41, H 7.47, N 6.59%. Single crystals of **I** and **II** suitable for X-ray diffraction analysis were selected from the samples resulting from the synthesis.

**Caution!** Perchlorate salts of metal complexes are potentially explosive and should be handled with care.

## 6. Refinement

Crystal data, data collection and structure refinement details are summarized in Table 4 ▸. The H atoms in **I** and **II** were placed in geometrically idealized positions and constrained to ride on their parent atoms, with C—H distances of 0.93, 0.96 and 0.97 Å (ring, methyl and methyl­ene H atoms, respectively), N—H distances of 0.98 Å, O—H distances of 0.82 Å (protonated carb­oxy­lic group) with *U*_iso_(H) values of 1.2*U*_eq_ or 1.5*U*_eq_ times those of the corresponding parent atoms. SQUEEZE calculations indicate the presence of nine water mol­ecules of crystallization per asymmetric unit of **II**.

## Supplementary Material

Crystal structure: contains datablock(s) I, II. DOI: 10.1107/S2056989024012337/hb8118sup1.cif

Structure factors: contains datablock(s) I. DOI: 10.1107/S2056989024012337/hb8118Isup2.hkl

Structure factors: contains datablock(s) II. DOI: 10.1107/S2056989024012337/hb8118IIsup3.hkl

CCDC references: 2373135, 2373134

Additional supporting information:  crystallographic information; 3D view; checkCIF report

## Figures and Tables

**Table 1:** Selected geometric parameters (Å, °)

	**I**	**II**
Ni1—N1	2.064 (2)	2.061 (4)
Ni1—N2	2.072 (2)	2.065 (4)
Ni2—N3	2.063 (2)	2.073 (4)
Ni2—N4	2.072 (2)	2.062 (4)
Ni1—O1	2.1625 (16)	2.191 (2)
Ni2—O2	2.1696 (16)	2.107 (3)
		
N1—Ni1—N2^i^	85.51 (9)	85.28 (19)
N1—Ni1—N2	94.49 (9)	94.72 (19)
N3—Ni2—N4^ii^	85.41 (9)	85.84 (19)
N3—Ni2—N4	94.59 (9)	94.16 (19)

Symmetry codes: (i) −*x*, −*y* + 1, −*z* + 1 in **I**[Chem scheme1] and −*x* + 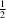, −*y* + 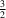, −*z* + 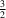 in **II**[Chem scheme1]; (ii) −*x* + 1, −*y* + 1, −*z* + 2 in **I**[Chem scheme1] and −*x* + 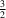, −*y* + 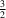, −*z* + 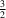 in **II**[Chem scheme1].

**Table 2:** Hydrogen-bond geometry (Å, °) for **I**[Chem scheme1]

*D*—H⋯*A*	*D*—H	H⋯*A*	*D*⋯*A*	*D*—H⋯*A*
N1—H1⋯O3	0.98	2.02	2.916 (3)	151
N2—H2⋯O4	0.98	2.05	2.960 (3)	154
N3—H3⋯O4	0.98	2.01	2.938 (3)	157
N4—H4⋯O3	0.98	2.04	2.946 (3)	153
C5—H5*A*⋯O3^i^	0.97	2.52	3.336 (4)	141
C6—H6*B*⋯O4^ii^	0.97	2.51	3.245 (3)	133

Symmetry codes: (i) 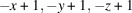; (ii) 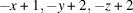.

**Table 3:** Hydrogen-bond geometry (Å, °) for **II**[Chem scheme1]

*D*—H⋯*A*	*D*—H	H⋯*A*	*D*⋯*A*	*D*—H⋯*A*
N1—H1⋯O4	0.98	2.32	3.113 (5)	138
N2—H2⋯O3	0.98	2.42	3.267 (4)	144
N4—H4⋯O3^i^	0.98	2.10	3.012 (5)	154
O5—H5⋯O4	0.82	1.79	2.597 (5)	169
O7—H7⋯O3^ii^	0.82	1.85	2.654 (5)	166

Symmetry codes: (i) 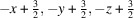; (ii) 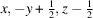.

**Table 4:** Experimental details

	**I**	**II**
Crystal data		
Chemical formula	[Ni(SO_4_)(C_10_H_24_N_4_)]	[Ni(SO_4_)(C_10_H_24_N_4_)]_2_·C_46_H_38_O_8_·18H_2_O
*M*_r_	355.10	1428.96
Crystal system, space group	Triclinic, *P* 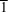	Monoclinic, *I*2/*a*
Temperature (K)	293	293
*a*, *b*, *c* (Å)	7.9935 (6), 8.3181 (6), 12.1998 (9)	12.1299 (5), 18.5163 (11), 35.621 (2)
α, β, γ (°)	108.308 (7), 102.767 (7), 99.145 (6)	90, 94.777 (4), 90
*V* (Å^3^)	727.88 (10)	7972.7 (8)
*Z*	2	4
Radiation type	Mo *K*α	Mo *K*α
μ (mm^−1^)	1.50	0.59
Crystal size (mm)	0.10 × 0.05 × 0.04	0.10 × 0.04 × 0.01

Data collection		
Diffractometer	Xcalibur, Eos	Xcalibur, Eos
Absorption correction	Multi-scan (*CrysAlis PRO*; Rigaku OD, 2022 ▸)	Multi-scan (*CrysAlis PRO*; Rigaku OD, 2022 ▸)
*T*_min_, *T*_max_	0.986, 1.000	0.920, 1.000
No. of measured, independent and observed [*I* > 2σ(*I*)] reflections	7381, 2965, 2356	20054, 8085, 3223
*R*_int_	0.036	0.086
(sin θ/λ)_max_ (Å^−1^)	0.625	0.625

Refinement		
*R*[*F*^2^ > 2σ(*F*^2^)], *wR*(*F*^2^), *S*	0.038, 0.080, 1.02	0.077, 0.123, 0.99
No. of reflections	2965	8085
No. of parameters	184	432
H-atom treatment	H-atom parameters constrained	H-atom parameters constrained
Δρ_max_, Δρ_min_ (e Å^−3^)	0.41, −0.41	0.25, −0.35

Computer programs: *CrysAlis PRO* (Rigaku OD, 2022 ▸), *SHELXT2018/2* (Sheldrick, 2015*a* ▸), *SHELXL2018/3* (Sheldrick, 2015*b* ▸), *Mercury* (Macrae *et al.*, 2020 ▸) and *publCIF* (Westrip, 2010 ▸).

## supplementary crystallographic information

### catena-Poly[[(1,4,8,11-tetraazacyclotetradecane-κ4N1,N4,N8,N11)nickel(II)]-µ2-sulfato-κ2O3:O4] (I) Crystal data

**Table:**

[Ni(SO_4_)(C_10_H_24_N_4_)]	*Z* = 2
*M**_r_* = 355.10	*F*(000) = 376
Triclinic, *P*1	*D*_x_ = 1.620 Mg m^−^^3^
*a* = 7.9935 (6) Å	Mo *K*α radiation, λ = 0.71073 Å
*b* = 8.3181 (6) Å	Cell parameters from 2779 reflections
*c* = 12.1998 (9) Å	θ = 2.6–27.9°
α = 108.308 (7)°	µ = 1.50 mm^−^^1^
β = 102.767 (7)°	*T* = 293 K
γ = 99.145 (6)°	Prism, clear light violet
*V* = 727.88 (10) Å^3^	0.10 × 0.05 × 0.04 mm

### catena-Poly[[(1,4,8,11-tetraazacyclotetradecane-κ4N1,N4,N8,N11)nickel(II)]-µ2-sulfato-κ2O3:O4] (I) Data collection

**Table:**

Xcalibur, Eos diffractometer	2965 independent reflections
Radiation source: fine-focus sealed X-ray tube, Enhance (Mo) X-ray Source	2356 reflections with *I* > 2σ(*I*)
Graphite monochromator	*R*_int_ = 0.036
Detector resolution: 16.1593 pixels mm^-1^	θ_max_ = 26.4°, θ_min_ = 1.8°
ω scans	*h* = −9→9
Absorption correction: multi-scan (CrysAlisPro; Rigaku OD, 2022)	*k* = −10→10
*T*_min_ = 0.986, *T*_max_ = 1.000	*l* = −15→15
7381 measured reflections	

### catena-Poly[[(1,4,8,11-tetraazacyclotetradecane-κ4N1,N4,N8,N11)nickel(II)]-µ2-sulfato-κ2O3:O4] (I) Refinement

**Table:**

Refinement on *F*^2^	Primary atom site location: dual
Least-squares matrix: full	Hydrogen site location: inferred from neighbouring sites
*R*[*F*^2^ > 2σ(*F*^2^)] = 0.038	H-atom parameters constrained
*wR*(*F*^2^) = 0.080	*w* = 1/[σ^2^(*F*_o_^2^) + (0.0285*P*)^2^] where *P* = (*F*_o_^2^ + 2*F*_c_^2^)/3
*S* = 1.02	(Δ/σ)_max_ = 0.001
2965 reflections	Δρ_max_ = 0.41 e Å^−^^3^
184 parameters	Δρ_min_ = −0.41 e Å^−^^3^
0 restraints	

### catena-Poly[[(1,4,8,11-tetraazacyclotetradecane-κ4N1,N4,N8,N11)nickel(II)]-µ2-sulfato-κ2O3:O4] (I) Special details

**Table:**

Geometry. All esds (except the esd in the dihedral angle between two l.s. planes) are estimated using the full covariance matrix. The cell esds are taken into account individually in the estimation of esds in distances, angles and torsion angles; correlations between esds in cell parameters are only used when they are defined by crystal symmetry. An approximate (isotropic) treatment of cell esds is used for estimating esds involving l.s. planes.

### catena-Poly[[(1,4,8,11-tetraazacyclotetradecane-κ4N1,N4,N8,N11)nickel(II)]-µ2-sulfato-κ2O3:O4] (I) Fractional atomic coordinates and isotropic or equivalent isotropic displacement parameters (Å^2^)

**Table:**

	*x*	*y*	*z*	*U*_iso_*/*U*_eq_	
Ni1	0.000000	0.500000	0.500000	0.01844 (14)	
Ni2	0.500000	0.500000	1.000000	0.01761 (14)	
S1	0.25415 (9)	0.52446 (9)	0.75638 (5)	0.02297 (18)	
O2	0.2854 (2)	0.4234 (2)	0.83542 (14)	0.0247 (4)	
O3	0.4087 (3)	0.5606 (4)	0.71535 (19)	0.0627 (8)	
O1	0.0987 (2)	0.4245 (2)	0.65152 (14)	0.0255 (5)	
O4	0.2186 (3)	0.6902 (3)	0.82444 (17)	0.0520 (7)	
N4	0.6885 (3)	0.5545 (3)	0.91572 (18)	0.0234 (5)	
H4	0.625381	0.552117	0.836403	0.028*	
N1	0.2431 (3)	0.5189 (3)	0.46653 (18)	0.0257 (5)	
H1	0.326732	0.513375	0.536522	0.031*	
N2	0.0549 (3)	0.7614 (3)	0.60765 (18)	0.0241 (5)	
H2	0.126128	0.775590	0.688102	0.029*	
N3	0.4696 (3)	0.7518 (3)	1.05788 (18)	0.0227 (5)	
H3	0.389541	0.764987	0.989536	0.027*	
C4	−0.1178 (4)	0.7948 (4)	0.6181 (3)	0.0375 (8)	
H4A	−0.181748	0.808385	0.545437	0.045*	
H4B	−0.100321	0.901524	0.685933	0.045*	
C5	0.2222 (4)	0.3575 (4)	0.3638 (2)	0.0342 (8)	
H5A	0.337616	0.339375	0.358732	0.041*	
H5B	0.160841	0.367627	0.289181	0.041*	
C10	0.3754 (4)	0.7608 (4)	1.1497 (2)	0.0316 (7)	
H10A	0.325008	0.861514	1.163556	0.038*	
H10B	0.456846	0.772294	1.225193	0.038*	
C7	0.7280 (4)	0.8752 (4)	1.0018 (3)	0.0354 (8)	
H7A	0.816441	0.983602	1.025167	0.043*	
H7B	0.642961	0.860916	0.926965	0.043*	
C6	0.6331 (4)	0.8916 (3)	1.0980 (3)	0.0322 (7)	
H6A	0.711889	0.887524	1.169491	0.039*	
H6B	0.603806	1.004011	1.119676	0.039*	
C3	0.1549 (4)	0.8829 (4)	0.5660 (2)	0.0330 (7)	
H3A	0.176117	1.001434	0.622139	0.040*	
H3B	0.084841	0.875543	0.487966	0.040*	
C2	0.3306 (4)	0.8428 (4)	0.5557 (3)	0.0377 (8)	
H2A	0.390275	0.829608	0.629642	0.045*	
H2B	0.403653	0.942128	0.549018	0.045*	
C9	0.7699 (4)	0.4053 (4)	0.8955 (2)	0.0301 (7)	
H9A	0.852602	0.415529	0.970157	0.036*	
H9B	0.834192	0.403040	0.836469	0.036*	
C8	0.8185 (4)	0.7247 (4)	0.9782 (2)	0.0314 (7)	
H8A	0.896344	0.739395	0.929486	0.038*	
H8B	0.890260	0.727149	1.054362	0.038*	
C1	0.3180 (4)	0.6798 (4)	0.4498 (3)	0.0360 (8)	
H1A	0.244293	0.684103	0.376506	0.043*	
H1B	0.435079	0.677907	0.440370	0.043*	

### catena-Poly[[(1,4,8,11-tetraazacyclotetradecane-κ4N1,N4,N8,N11)nickel(II)]-µ2-sulfato-κ2O3:O4] (I) Atomic displacement parameters (Å^2^)

**Table:**

	*U*^11^	*U*^22^	*U*^33^	*U*^12^	*U*^13^	*U*^23^
Ni1	0.0161 (3)	0.0253 (3)	0.0139 (2)	0.0052 (2)	0.00150 (19)	0.0091 (2)
Ni2	0.0177 (3)	0.0196 (3)	0.0146 (2)	0.0044 (2)	0.00097 (19)	0.0076 (2)
S1	0.0191 (4)	0.0317 (4)	0.0166 (3)	0.0020 (3)	−0.0022 (3)	0.0138 (3)
O2	0.0273 (11)	0.0258 (10)	0.0180 (9)	0.0037 (9)	−0.0044 (8)	0.0129 (8)
O3	0.0203 (12)	0.136 (2)	0.0444 (14)	0.0038 (13)	0.0024 (10)	0.0619 (15)
O1	0.0251 (11)	0.0296 (11)	0.0165 (9)	0.0023 (9)	−0.0044 (8)	0.0104 (8)
O4	0.0816 (18)	0.0276 (12)	0.0285 (12)	0.0197 (12)	−0.0156 (12)	0.0046 (10)
N4	0.0214 (13)	0.0282 (13)	0.0193 (11)	0.0051 (10)	0.0006 (10)	0.0111 (10)
N1	0.0221 (13)	0.0386 (14)	0.0181 (12)	0.0080 (11)	0.0036 (10)	0.0138 (11)
N2	0.0276 (14)	0.0272 (13)	0.0167 (11)	0.0078 (11)	0.0007 (10)	0.0106 (10)
N3	0.0233 (13)	0.0203 (12)	0.0197 (11)	0.0052 (10)	−0.0020 (10)	0.0065 (10)
C4	0.040 (2)	0.0401 (19)	0.0343 (17)	0.0225 (16)	0.0109 (15)	0.0104 (15)
C5	0.0279 (18)	0.050 (2)	0.0317 (17)	0.0179 (16)	0.0165 (14)	0.0151 (15)
C10	0.0370 (19)	0.0306 (17)	0.0246 (15)	0.0150 (14)	0.0076 (13)	0.0039 (13)
C7	0.0340 (19)	0.0276 (16)	0.0391 (18)	−0.0032 (13)	−0.0011 (14)	0.0179 (14)
C6	0.0355 (19)	0.0190 (15)	0.0340 (17)	0.0024 (13)	−0.0006 (14)	0.0085 (13)
C3	0.043 (2)	0.0235 (16)	0.0248 (16)	0.0008 (14)	0.0003 (14)	0.0089 (13)
C2	0.0333 (19)	0.0409 (19)	0.0334 (17)	−0.0101 (15)	0.0016 (14)	0.0213 (15)
C9	0.0266 (17)	0.0396 (18)	0.0251 (15)	0.0134 (14)	0.0077 (13)	0.0104 (14)
C8	0.0240 (17)	0.0365 (18)	0.0302 (16)	−0.0023 (13)	0.0021 (13)	0.0160 (14)
C1	0.0240 (17)	0.058 (2)	0.0313 (16)	0.0046 (15)	0.0089 (13)	0.0247 (16)

### catena-Poly[[(1,4,8,11-tetraazacyclotetradecane-κ4N1,N4,N8,N11)nickel(II)]-µ2-sulfato-κ2O3:O4] (I) Geometric parameters (Å, º)

**Table:**

Ni1—O1^i^	2.1625 (16)	N3—C6	1.475 (4)
Ni1—O1	2.1625 (16)	C4—H4A	0.9700
Ni1—N1	2.064 (2)	C4—H4B	0.9700
Ni1—N1^i^	2.064 (2)	C4—C5^i^	1.507 (4)
Ni1—N2	2.072 (2)	C5—H5A	0.9700
Ni1—N2^i^	2.072 (2)	C5—H5B	0.9700
Ni2—O2^ii^	2.1696 (16)	C10—H10A	0.9700
Ni2—O2	2.1696 (16)	C10—H10B	0.9700
Ni2—N4^ii^	2.072 (2)	C10—C9^ii^	1.514 (4)
Ni2—N4	2.072 (2)	C7—H7A	0.9700
Ni2—N3^ii^	2.063 (2)	C7—H7B	0.9700
Ni2—N3	2.063 (2)	C7—C6	1.516 (4)
S1—O2	1.4722 (17)	C7—C8	1.526 (4)
S1—O3	1.455 (2)	C6—H6A	0.9700
S1—O1	1.4742 (17)	C6—H6B	0.9700
S1—O4	1.477 (2)	C3—H3A	0.9700
N4—H4	0.9800	C3—H3B	0.9700
N4—C9	1.470 (3)	C3—C2	1.517 (4)
N4—C8	1.473 (3)	C2—H2A	0.9700
N1—H1	0.9800	C2—H2B	0.9700
N1—C5	1.476 (3)	C2—C1	1.522 (4)
N1—C1	1.471 (4)	C9—H9A	0.9700
N2—H2	0.9800	C9—H9B	0.9700
N2—C4	1.475 (3)	C8—H8A	0.9700
N2—C3	1.470 (3)	C8—H8B	0.9700
N3—H3	0.9800	C1—H1A	0.9700
N3—C10	1.471 (3)	C1—H1B	0.9700
			
O1^i^—Ni1—O1	180.0	C6—N3—H3	107.1
N1—Ni1—O1	90.53 (7)	N2—C4—H4A	110.0
N1^i^—Ni1—O1	89.47 (7)	N2—C4—H4B	110.0
N1^i^—Ni1—O1^i^	90.53 (7)	N2—C4—C5^i^	108.6 (2)
N1—Ni1—O1^i^	89.47 (7)	H4A—C4—H4B	108.3
N1^i^—Ni1—N1	180.0	C5^i^—C4—H4A	110.0
N1—Ni1—N2	94.49 (9)	C5^i^—C4—H4B	110.0
N1—Ni1—N2^i^	85.51 (9)	N1—C5—C4^i^	108.9 (2)
N1^i^—Ni1—N2	85.51 (9)	N1—C5—H5A	109.9
N1^i^—Ni1—N2^i^	94.49 (9)	N1—C5—H5B	109.9
N2^i^—Ni1—O1	87.02 (7)	C4^i^—C5—H5A	109.9
N2—Ni1—O1	92.98 (7)	C4^i^—C5—H5B	109.9
N2^i^—Ni1—O1^i^	92.98 (7)	H5A—C5—H5B	108.3
N2—Ni1—O1^i^	87.02 (7)	N3—C10—H10A	110.1
N2—Ni1—N2^i^	180.00 (7)	N3—C10—H10B	110.1
O2—Ni2—O2^ii^	180.0	N3—C10—C9^ii^	108.2 (2)
N4^ii^—Ni2—O2^ii^	92.22 (7)	H10A—C10—H10B	108.4
N4—Ni2—O2^ii^	87.78 (7)	C9^ii^—C10—H10A	110.1
N4^ii^—Ni2—O2	87.78 (7)	C9^ii^—C10—H10B	110.1
N4—Ni2—O2	92.22 (7)	H7A—C7—H7B	107.5
N4—Ni2—N4^ii^	180.0	C6—C7—H7A	108.5
N3^ii^—Ni2—O2	87.88 (7)	C6—C7—H7B	108.5
N3—Ni2—O2^ii^	87.87 (7)	C6—C7—C8	115.2 (2)
N3^ii^—Ni2—O2^ii^	92.12 (7)	C8—C7—H7A	108.5
N3—Ni2—O2	92.13 (7)	C8—C7—H7B	108.5
N3^ii^—Ni2—N4	85.41 (9)	N3—C6—C7	112.4 (2)
N3—Ni2—N4	94.59 (9)	N3—C6—H6A	109.1
N3—Ni2—N4^ii^	85.41 (9)	N3—C6—H6B	109.1
N3^ii^—Ni2—N4^ii^	94.58 (9)	C7—C6—H6A	109.1
N3—Ni2—N3^ii^	180.00 (11)	C7—C6—H6B	109.1
O2—S1—O1	109.61 (11)	H6A—C6—H6B	107.8
O2—S1—O4	108.92 (11)	N2—C3—H3A	109.2
O3—S1—O2	109.99 (12)	N2—C3—H3B	109.2
O3—S1—O1	109.64 (12)	N2—C3—C2	111.9 (2)
O3—S1—O4	109.85 (16)	H3A—C3—H3B	107.9
O1—S1—O4	108.81 (11)	C2—C3—H3A	109.2
S1—O2—Ni2	126.36 (11)	C2—C3—H3B	109.2
S1—O1—Ni1	126.17 (11)	C3—C2—H2A	108.5
Ni2—N4—H4	107.4	C3—C2—H2B	108.5
C9—N4—Ni2	104.98 (17)	C3—C2—C1	115.1 (2)
C9—N4—H4	107.4	H2A—C2—H2B	107.5
C9—N4—C8	113.3 (2)	C1—C2—H2A	108.5
C8—N4—Ni2	116.05 (17)	C1—C2—H2B	108.5
C8—N4—H4	107.4	N4—C9—C10^ii^	108.4 (2)
Ni1—N1—H1	106.9	N4—C9—H9A	110.0
C5—N1—Ni1	105.39 (17)	N4—C9—H9B	110.0
C5—N1—H1	106.9	C10^ii^—C9—H9A	110.0
C1—N1—Ni1	116.45 (17)	C10^ii^—C9—H9B	110.0
C1—N1—H1	106.9	H9A—C9—H9B	108.4
C1—N1—C5	113.8 (2)	N4—C8—C7	111.5 (2)
Ni1—N2—H2	107.5	N4—C8—H8A	109.3
C4—N2—Ni1	105.35 (17)	N4—C8—H8B	109.3
C4—N2—H2	107.5	C7—C8—H8A	109.3
C3—N2—Ni1	114.87 (16)	C7—C8—H8B	109.3
C3—N2—H2	107.5	H8A—C8—H8B	108.0
C3—N2—C4	113.8 (2)	N1—C1—C2	112.0 (2)
Ni2—N3—H3	107.1	N1—C1—H1A	109.2
C10—N3—Ni2	105.45 (16)	N1—C1—H1B	109.2
C10—N3—H3	107.1	C2—C1—H1A	109.2
C10—N3—C6	114.1 (2)	C2—C1—H1B	109.2
C6—N3—Ni2	115.61 (16)	H1A—C1—H1B	107.9
			
Ni1—N1—C5—C4^i^	−41.9 (2)	O4—S1—O1—Ni1	65.05 (16)
Ni1—N1—C1—C2	54.9 (3)	N2—C3—C2—C1	73.0 (3)
Ni1—N2—C4—C5^i^	41.8 (2)	C4—N2—C3—C2	−179.6 (2)
Ni1—N2—C3—C2	−58.0 (2)	C5—N1—C1—C2	177.9 (2)
Ni2—N4—C9—C10^ii^	43.1 (2)	C10—N3—C6—C7	178.8 (2)
Ni2—N4—C8—C7	−56.2 (3)	C6—N3—C10—C9^ii^	−170.5 (2)
Ni2—N3—C10—C9^ii^	−42.5 (2)	C6—C7—C8—N4	71.5 (3)
Ni2—N3—C6—C7	56.2 (3)	C3—N2—C4—C5^i^	168.5 (2)
O2—S1—O1—Ni1	−175.93 (11)	C3—C2—C1—N1	−70.7 (3)
O3—S1—O2—Ni2	57.29 (17)	C9—N4—C8—C7	−177.7 (2)
O3—S1—O1—Ni1	−55.11 (17)	C8—N4—C9—C10^ii^	170.7 (2)
O1—S1—O2—Ni2	177.90 (11)	C8—C7—C6—N3	−71.8 (3)
O4—S1—O2—Ni2	−63.16 (16)	C1—N1—C5—C4^i^	−170.7 (2)

Symmetry codes: (i) −*x*, −*y*+1, −*z*+1; (ii) −*x*+1, −*y*+1, −*z*+2.

### catena-Poly[[(1,4,8,11-tetraazacyclotetradecane-κ4N1,N4,N8,N11)nickel(II)]-µ2-sulfato-κ2O3:O4] (I) Hydrogen-bond geometry (Å, º)

**Table:**

*D*—H···*A*	*D*—H	H···*A*	*D*···*A*	*D*—H···*A*
N1—H1···O3	0.98	2.02	2.916 (3)	151
N2—H2···O4	0.98	2.05	2.960 (3)	154
N3—H3···O4	0.98	2.01	2.938 (3)	157
N4—H4···O3	0.98	2.04	2.946 (3)	153
C5—H5*A*···O3^iii^	0.97	2.52	3.336 (4)	141
C6—H6*B*···O4^iv^	0.97	2.51	3.245 (3)	133

Symmetry codes: (iii) −*x*+1, −*y*+1, −*z*+1; (iv) −*x*+1, −*y*+2, −*z*+2.

### catena-Poly[[[(1,4,8,11-tetraazacyclotetradecane-κ4N1,N4,N8,N11)nickel(II)]-µ2-sulfato-κ2O3:O4] hemi[4,4',4'',4'''-(2,2',4,4',6,6'-hexamethyl-[1,1'-biphenyl]-3,3',5,5'-tetrayl)tetrabenzoic acid] nonahydrate] (II) Crystal data

**Table:**

[Ni(SO_4_)(C_10_H_24_N_4_)]_2_·C_46_H_38_O_8_·18H_2_O	*F*(000) = 3016
*M**_r_* = 1428.96	*D*_x_ = 1.190 Mg m^−^^3^
Monoclinic, *I*2/*a*	Mo *K*α radiation, λ = 0.71073 Å
*a* = 12.1299 (5) Å	Cell parameters from 2340 reflections
*b* = 18.5163 (11) Å	θ = 2.0–21.1°
*c* = 35.621 (2) Å	µ = 0.59 mm^−^^1^
β = 94.777 (4)°	*T* = 293 K
*V* = 7972.7 (8) Å^3^	Plate, clear light colourless
*Z* = 4	0.10 × 0.04 × 0.01 mm

### catena-Poly[[[(1,4,8,11-tetraazacyclotetradecane-κ4N1,N4,N8,N11)nickel(II)]-µ2-sulfato-κ2O3:O4] hemi[4,4',4'',4'''-(2,2',4,4',6,6'-hexamethyl-[1,1'-biphenyl]-3,3',5,5'-tetrayl)tetrabenzoic acid] nonahydrate] (II) Data collection

**Table:**

Xcalibur, Eos diffractometer	8085 independent reflections
Radiation source: fine-focus sealed X-ray tube, Enhance (Mo) X-ray Source	3223 reflections with *I* > 2σ(*I*)
Graphite monochromator	*R*_int_ = 0.086
Detector resolution: 16.1593 pixels mm^-1^	θ_max_ = 26.4°, θ_min_ = 2.0°
ω scans	*h* = −15→15
Absorption correction: multi-scan (CrysAlisPro; Rigaku OD, 2022)	*k* = −15→23
*T*_min_ = 0.920, *T*_max_ = 1.000	*l* = −23→44
20054 measured reflections	

### catena-Poly[[[(1,4,8,11-tetraazacyclotetradecane-κ4N1,N4,N8,N11)nickel(II)]-µ2-sulfato-κ2O3:O4] hemi[4,4',4'',4'''-(2,2',4,4',6,6'-hexamethyl-[1,1'-biphenyl]-3,3',5,5'-tetrayl)tetrabenzoic acid] nonahydrate] (II) Refinement

**Table:**

Refinement on *F*^2^	Primary atom site location: dual
Least-squares matrix: full	Hydrogen site location: mixed
*R*[*F*^2^ > 2σ(*F*^2^)] = 0.077	H-atom parameters constrained
*wR*(*F*^2^) = 0.123	*w* = 1/[σ^2^(*F*_o_^2^) + (0.0206*P*)^2^] where *P* = (*F*_o_^2^ + 2*F*_c_^2^)/3
*S* = 0.99	(Δ/σ)_max_ < 0.001
8085 reflections	Δρ_max_ = 0.25 e Å^−^^3^
432 parameters	Δρ_min_ = −0.35 e Å^−^^3^
0 restraints	

### catena-Poly[[[(1,4,8,11-tetraazacyclotetradecane-κ4N1,N4,N8,N11)nickel(II)]-µ2-sulfato-κ2O3:O4] hemi[4,4',4'',4'''-(2,2',4,4',6,6'-hexamethyl-[1,1'-biphenyl]-3,3',5,5'-tetrayl)tetrabenzoic acid] nonahydrate] (II) Special details

**Table:**

Geometry. All esds (except the esd in the dihedral angle between two l.s. planes) are estimated using the full covariance matrix. The cell esds are taken into account individually in the estimation of esds in distances, angles and torsion angles; correlations between esds in cell parameters are only used when they are defined by crystal symmetry. An approximate (isotropic) treatment of cell esds is used for estimating esds involving l.s. planes.

### catena-Poly[[[(1,4,8,11-tetraazacyclotetradecane-κ4N1,N4,N8,N11)nickel(II)]-µ2-sulfato-κ2O3:O4] hemi[4,4',4'',4'''-(2,2',4,4',6,6'-hexamethyl-[1,1'-biphenyl]-3,3',5,5'-tetrayl)tetrabenzoic acid] nonahydrate] (II) Fractional atomic coordinates and isotropic or equivalent isotropic displacement parameters (Å^2^)

**Table:**

	*x*	*y*	*z*	*U*_iso_*/*U*_eq_	Occ. (<1)
Ni1	0.250000	0.750000	0.750000	0.0562 (3)	
Ni2	0.750000	0.750000	0.750000	0.0628 (3)	
S1	0.50054 (10)	0.67856 (9)	0.75649 (4)	0.0648 (5)	
O3	0.5194 (2)	0.6766 (2)	0.79768 (10)	0.0784 (12)	
O2	0.60695 (19)	0.68618 (17)	0.73957 (8)	0.0637 (10)	
N3	0.8460 (3)	0.6578 (3)	0.74746 (15)	0.0723 (14)	
H3	0.923074	0.673461	0.747240	0.087*	
O1	0.4286 (2)	0.74068 (18)	0.74571 (9)	0.0633 (10)	
O4	0.4467 (2)	0.6099 (2)	0.74284 (10)	0.0839 (13)	
N1	0.2244 (3)	0.6743 (3)	0.70776 (12)	0.0646 (13)	
H1	0.296188	0.651709	0.704761	0.078*	
N2	0.2493 (3)	0.6758 (3)	0.79323 (12)	0.0724 (15)	
H2	0.322725	0.653431	0.796249	0.087*	
N4	0.7455 (3)	0.7698 (3)	0.69294 (12)	0.0752 (15)	
H4	0.815571	0.792987	0.688167	0.090*	
C30	0.6311 (4)	0.1028 (2)	0.43972 (16)	0.0549 (15)	
O6	0.3958 (3)	0.6092 (2)	0.64754 (11)	0.1048 (15)	
C11	0.4785 (4)	0.5740 (3)	0.6510 (2)	0.0739 (19)	
O8	0.3879 (3)	−0.0478 (2)	0.34768 (11)	0.0993 (15)	
C18	0.6922 (3)	0.4520 (2)	0.52586 (16)	0.0565 (15)	
C15	0.6387 (4)	0.4891 (3)	0.55700 (18)	0.0578 (16)	
O5	0.5273 (3)	0.5560 (3)	0.68365 (12)	0.1085 (17)	
H5	0.494848	0.574604	0.700454	0.163*	
C35	0.6920 (4)	0.0749 (3)	0.41216 (17)	0.0641 (17)	
H35	0.767412	0.084687	0.413391	0.077*	
O7	0.5501 (4)	−0.0438 (2)	0.32448 (15)	0.141 (2)	
H7	0.544646	−0.087006	0.319664	0.212*	
C24	0.750000	0.2565 (4)	0.500000	0.061 (2)	
C10	0.8358 (4)	0.6201 (4)	0.7837 (2)	0.099 (2)	
H10A	0.894279	0.584556	0.787911	0.118*	
H10B	0.765234	0.595217	0.783070	0.118*	
C12	0.5369 (4)	0.5458 (3)	0.61875 (18)	0.0632 (17)	
C33	0.5339 (4)	0.0202 (2)	0.37979 (15)	0.0549 (15)	
C32	0.4706 (4)	0.0485 (3)	0.40693 (17)	0.0667 (17)	
H32	0.394832	0.040027	0.405222	0.080*	
C29	0.750000	0.0240 (3)	0.500000	0.081 (3)	
H29A	0.682200	0.006673	0.508860	0.122*	0.5
H29B	0.811420	0.006673	0.516270	0.122*	0.5
H29C	0.756370	0.006673	0.474860	0.122*	0.5
C25	0.6867 (4)	0.2198 (3)	0.47185 (17)	0.0632 (16)	
C31	0.5178 (4)	0.0887 (3)	0.43612 (17)	0.0724 (18)	
H31	0.473456	0.107060	0.453963	0.087*	
C21	0.6887 (4)	0.3754 (2)	0.52488 (16)	0.0598 (16)	
C2	0.1792 (4)	0.5761 (3)	0.7521 (2)	0.102 (3)	
H2A	0.134603	0.532745	0.752629	0.123*	
H2B	0.255789	0.561034	0.751981	0.123*	
C14	0.5408 (4)	0.5268 (3)	0.55178 (17)	0.0761 (18)	
H14	0.507398	0.533655	0.527591	0.091*	
C19	0.750000	0.4897 (4)	0.500000	0.059 (2)	
C7	0.8269 (4)	0.6526 (4)	0.67781 (18)	0.100 (2)	
H7A	0.827753	0.618110	0.657331	0.120*	
H7B	0.896294	0.678843	0.679027	0.120*	
C1	0.1470 (4)	0.6156 (3)	0.71504 (19)	0.091 (2)	
H1A	0.145034	0.581271	0.694445	0.109*	
H1B	0.073304	0.635565	0.715824	0.109*	
C34	0.6463 (4)	0.0332 (3)	0.38290 (16)	0.0628 (16)	
H34	0.690847	0.014029	0.365352	0.075*	
C26	0.6144 (4)	0.2602 (3)	0.44165 (17)	0.093 (2)	
H26A	0.628524	0.311130	0.443719	0.140*	
H26B	0.537924	0.251010	0.444999	0.140*	
H26C	0.630924	0.243690	0.417199	0.140*	
C28	0.750000	0.1063 (3)	0.500000	0.053 (2)	
C36	0.4816 (5)	−0.0268 (3)	0.34931 (17)	0.0710 (18)	
C6	0.8214 (4)	0.6113 (3)	0.7142 (2)	0.093 (2)	
H6A	0.748034	0.590791	0.714947	0.112*	
H6B	0.874045	0.571789	0.714897	0.112*	
C27	0.6889 (4)	0.1440 (3)	0.47161 (16)	0.0581 (15)	
C23	0.750000	0.3381 (3)	0.500000	0.059 (2)	
C4	0.2352 (4)	0.7191 (4)	0.82776 (17)	0.092 (2)	
H4A	0.257107	0.690753	0.850039	0.111*	
H4B	0.158213	0.732707	0.828578	0.111*	
C13	0.4922 (4)	0.5543 (3)	0.5823 (2)	0.077 (2)	
H13	0.426278	0.579840	0.578109	0.092*	
C17	0.6369 (4)	0.5105 (3)	0.62374 (17)	0.087 (2)	
H17	0.671219	0.504953	0.647893	0.105*	
C16	0.6867 (4)	0.4833 (3)	0.5933 (2)	0.099 (2)	
H16	0.754840	0.460414	0.597436	0.118*	
C5	0.1940 (4)	0.7144 (4)	0.67308 (16)	0.089 (2)	
H5A	0.116564	0.728053	0.672049	0.106*	
H5B	0.204755	0.684329	0.651400	0.106*	
C3	0.1672 (4)	0.6173 (4)	0.7876 (2)	0.099 (2)	
H3A	0.093417	0.637773	0.786818	0.119*	
H3B	0.175758	0.584418	0.808869	0.119*	
C9	0.6560 (4)	0.8245 (4)	0.68479 (18)	0.100 (2)	
H9A	0.584152	0.801136	0.683389	0.120*	
H9B	0.664611	0.847849	0.660847	0.120*	
C22	0.6150 (4)	0.3346 (3)	0.55038 (16)	0.096 (2)	
H22A	0.652519	0.330370	0.575101	0.144*	
H22B	0.598969	0.287350	0.540311	0.144*	
H22C	0.547179	0.360810	0.551891	0.144*	
C8	0.7330 (4)	0.7059 (4)	0.66839 (17)	0.099 (2)	
H8A	0.733069	0.720682	0.642270	0.119*	
H8B	0.662777	0.682610	0.671625	0.119*	
C20	0.750000	0.5710 (3)	0.500000	0.086 (3)	
H20A	0.675440	0.588307	0.500220	0.129*	0.5
H20B	0.781320	0.588307	0.477810	0.129*	0.5
H20C	0.793240	0.588307	0.521970	0.129*	0.5

### catena-Poly[[[(1,4,8,11-tetraazacyclotetradecane-κ4N1,N4,N8,N11)nickel(II)]-µ2-sulfato-κ2O3:O4] hemi[4,4',4'',4'''-(2,2',4,4',6,6'-hexamethyl-[1,1'-biphenyl]-3,3',5,5'-tetrayl)tetrabenzoic acid] nonahydrate] (II) Atomic displacement parameters (Å^2^)

**Table:**

	*U*^11^	*U*^22^	*U*^33^	*U*^12^	*U*^13^	*U*^23^
Ni1	0.0282 (5)	0.1032 (8)	0.0381 (6)	0.0154 (5)	0.0081 (4)	0.0213 (6)
Ni2	0.0268 (5)	0.1156 (8)	0.0474 (7)	0.0160 (5)	0.0113 (4)	0.0303 (7)
S1	0.0272 (6)	0.1131 (13)	0.0562 (11)	0.0168 (8)	0.0155 (6)	0.0301 (11)
O3	0.047 (2)	0.140 (3)	0.049 (3)	0.029 (2)	0.0138 (17)	0.044 (3)
O2	0.0217 (16)	0.117 (3)	0.055 (3)	0.0142 (16)	0.0150 (15)	0.018 (2)
N3	0.030 (2)	0.122 (4)	0.066 (4)	0.014 (2)	0.010 (2)	0.023 (4)
O1	0.0313 (16)	0.104 (3)	0.055 (2)	0.0224 (17)	0.0082 (15)	0.026 (2)
O4	0.040 (2)	0.105 (3)	0.110 (4)	0.005 (2)	0.028 (2)	0.009 (3)
N1	0.032 (2)	0.117 (4)	0.046 (3)	0.022 (2)	0.010 (2)	−0.001 (3)
N2	0.030 (2)	0.132 (4)	0.057 (4)	0.025 (3)	0.015 (2)	0.042 (3)
N4	0.037 (2)	0.139 (5)	0.051 (4)	0.010 (3)	0.014 (2)	0.029 (3)
C30	0.068 (4)	0.033 (3)	0.066 (5)	0.004 (3)	0.020 (3)	0.004 (3)
O6	0.065 (3)	0.143 (4)	0.108 (4)	0.053 (2)	0.011 (2)	−0.008 (3)
C11	0.047 (4)	0.084 (5)	0.092 (6)	0.012 (3)	0.015 (4)	−0.009 (4)
O8	0.051 (2)	0.133 (3)	0.116 (4)	−0.020 (2)	0.025 (2)	−0.044 (3)
C18	0.041 (3)	0.044 (3)	0.085 (5)	0.009 (2)	0.010 (3)	−0.007 (3)
C15	0.045 (3)	0.048 (3)	0.082 (5)	−0.002 (3)	0.011 (3)	0.004 (4)
O5	0.075 (3)	0.167 (4)	0.084 (4)	0.057 (3)	0.010 (3)	−0.005 (4)
C35	0.056 (3)	0.059 (4)	0.079 (5)	−0.010 (3)	0.015 (3)	0.000 (4)
O7	0.103 (3)	0.163 (4)	0.172 (5)	−0.076 (3)	0.096 (3)	−0.115 (4)
C24	0.069 (5)	0.032 (5)	0.086 (8)	0.000	0.026 (5)	0.000
C10	0.047 (4)	0.144 (7)	0.105 (7)	0.021 (4)	0.008 (4)	0.048 (6)
C12	0.042 (3)	0.068 (4)	0.081 (5)	0.012 (3)	0.009 (3)	0.004 (4)
C33	0.049 (3)	0.052 (3)	0.067 (5)	−0.001 (2)	0.021 (3)	−0.003 (3)
C32	0.043 (3)	0.072 (4)	0.087 (5)	−0.004 (3)	0.018 (3)	−0.019 (4)
C29	0.116 (6)	0.044 (5)	0.081 (7)	0.000	−0.010 (5)	0.000
C25	0.072 (4)	0.037 (4)	0.082 (5)	0.002 (3)	0.012 (3)	0.010 (3)
C31	0.062 (4)	0.073 (4)	0.086 (5)	0.003 (3)	0.032 (3)	−0.015 (4)
C21	0.055 (3)	0.042 (3)	0.086 (5)	−0.006 (3)	0.024 (3)	0.004 (3)
C2	0.042 (4)	0.105 (6)	0.161 (9)	0.005 (3)	0.019 (4)	0.025 (6)
C14	0.052 (3)	0.097 (4)	0.079 (5)	0.020 (3)	0.003 (3)	−0.020 (4)
C19	0.041 (4)	0.041 (5)	0.095 (7)	0.000	0.013 (4)	0.000
C7	0.071 (4)	0.156 (6)	0.077 (6)	−0.007 (4)	0.027 (4)	−0.004 (5)
C1	0.043 (3)	0.121 (6)	0.111 (7)	−0.003 (4)	0.011 (4)	−0.022 (5)
C34	0.054 (3)	0.057 (4)	0.082 (5)	−0.006 (3)	0.030 (3)	0.007 (3)
C26	0.105 (5)	0.053 (4)	0.121 (6)	0.009 (3)	0.000 (4)	0.005 (4)
C28	0.083 (5)	0.010 (4)	0.068 (7)	0.000	0.013 (4)	0.000
C36	0.066 (4)	0.077 (4)	0.074 (5)	−0.014 (3)	0.029 (4)	−0.022 (4)
C6	0.040 (3)	0.134 (6)	0.107 (7)	0.011 (3)	0.018 (4)	0.011 (6)
C27	0.055 (3)	0.045 (4)	0.074 (5)	0.000 (3)	0.008 (3)	−0.001 (3)
C23	0.050 (4)	0.028 (5)	0.099 (8)	0.000	0.014 (4)	0.000
C4	0.059 (4)	0.168 (7)	0.054 (5)	0.046 (4)	0.027 (3)	0.041 (5)
C13	0.049 (4)	0.081 (4)	0.100 (6)	0.027 (3)	0.008 (4)	0.004 (4)
C17	0.058 (3)	0.121 (5)	0.082 (5)	0.040 (3)	0.004 (3)	−0.005 (4)
C16	0.055 (4)	0.139 (6)	0.103 (6)	0.046 (4)	0.016 (4)	−0.015 (5)
C5	0.064 (4)	0.159 (7)	0.043 (4)	0.039 (4)	0.008 (3)	0.002 (5)
C3	0.050 (4)	0.129 (6)	0.123 (7)	0.015 (4)	0.032 (4)	0.057 (5)
C9	0.053 (4)	0.178 (7)	0.067 (5)	0.018 (4)	0.002 (3)	0.064 (5)
C22	0.103 (5)	0.064 (4)	0.129 (6)	−0.008 (3)	0.051 (4)	0.017 (4)
C8	0.057 (4)	0.181 (7)	0.062 (5)	0.000 (4)	0.013 (3)	0.021 (5)
C20	0.111 (6)	0.019 (4)	0.135 (9)	0.000	0.055 (5)	0.000

### catena-Poly[[[(1,4,8,11-tetraazacyclotetradecane-κ4N1,N4,N8,N11)nickel(II)]-µ2-sulfato-κ2O3:O4] hemi[4,4',4'',4'''-(2,2',4,4',6,6'-hexamethyl-[1,1'-biphenyl]-3,3',5,5'-tetrayl)tetrabenzoic acid] nonahydrate] (II) Geometric parameters (Å, º)

**Table:**

Ni1—O1	2.191 (2)	C33—C36	1.491 (6)
Ni1—O1^i^	2.191 (2)	C32—H32	0.9300
Ni1—N1	2.061 (4)	C32—C31	1.365 (6)
Ni1—N1^i^	2.061 (4)	C29—H29A	0.9600
Ni1—N2	2.065 (4)	C29—H29B	0.9600
Ni1—N2^i^	2.065 (4)	C29—H29C	0.9602
Ni2—O2	2.107 (3)	C29—C28	1.524 (8)
Ni2—O2^ii^	2.107 (3)	C25—C26	1.526 (6)
Ni2—N3^ii^	2.073 (4)	C25—C27	1.403 (6)
Ni2—N3	2.073 (4)	C31—H31	0.9300
Ni2—N4	2.062 (4)	C21—C23	1.388 (5)
Ni2—N4^ii^	2.062 (4)	C21—C22	1.526 (6)
S1—O3	1.466 (3)	C2—H2A	0.9700
S1—O2	1.476 (3)	C2—H2B	0.9700
S1—O1	1.476 (3)	C2—C1	1.530 (7)
S1—O4	1.491 (4)	C2—C3	1.496 (7)
N3—H3	0.9800	C14—H14	0.9300
N3—C10	1.481 (6)	C14—C13	1.378 (7)
N3—C6	1.476 (6)	C19—C20	1.506 (8)
N1—H1	0.9800	C7—H7A	0.9700
N1—C1	1.473 (6)	C7—H7B	0.9700
N1—C5	1.462 (6)	C7—C6	1.510 (7)
N2—H2	0.9800	C7—C8	1.523 (7)
N2—C4	1.490 (6)	C1—H1A	0.9700
N2—C3	1.473 (6)	C1—H1B	0.9700
N4—H4	0.9800	C34—H34	0.9300
N4—C9	1.495 (6)	C26—H26A	0.9599
N4—C8	1.472 (6)	C26—H26B	0.9601
C30—C35	1.377 (6)	C26—H26C	0.9600
C30—C31	1.395 (6)	C28—C27^iii^	1.391 (5)
C30—C27	1.494 (7)	C28—C27	1.391 (5)
O6—C11	1.194 (5)	C6—H6A	0.9700
C11—O5	1.305 (6)	C6—H6B	0.9700
C11—C12	1.492 (7)	C4—H4A	0.9700
O8—C36	1.198 (5)	C4—H4B	0.9700
C18—C15	1.498 (6)	C4—C5^i^	1.503 (7)
C18—C21	1.419 (6)	C13—H13	0.9300
C18—C19	1.391 (5)	C17—H17	0.9300
C15—C14	1.377 (6)	C17—C16	1.380 (7)
C15—C16	1.377 (7)	C16—H16	0.9300
O5—H5	0.8200	C5—H5A	0.9700
C35—H35	0.9300	C5—H5B	0.9700
C35—C34	1.376 (6)	C3—H3A	0.9700
O7—H7	0.8200	C3—H3B	0.9700
O7—C36	1.302 (5)	C9—H9A	0.9700
C24—C25^iii^	1.389 (6)	C9—H9B	0.9700
C24—C25	1.389 (6)	C22—H22A	0.9602
C24—C23	1.510 (9)	C22—H22B	0.9600
C10—H10A	0.9700	C22—H22C	0.9601
C10—H10B	0.9700	C8—H8A	0.9700
C10—C9^ii^	1.519 (7)	C8—H8B	0.9700
C12—C13	1.372 (7)	C20—H20A	0.9600
C12—C17	1.376 (6)	C20—H20B	0.9599
C33—C32	1.386 (6)	C20—H20C	0.9600
C33—C34	1.381 (5)		
			
O1—Ni1—O1^i^	180.0	C32—C31—C30	121.3 (5)
N1^i^—Ni1—O1	90.85 (13)	C32—C31—H31	119.4
N1—Ni1—O1^i^	90.85 (13)	C18—C21—C22	119.8 (4)
N1—Ni1—O1	89.15 (13)	C23—C21—C18	119.8 (5)
N1^i^—Ni1—O1^i^	89.15 (13)	C23—C21—C22	120.3 (4)
N1—Ni1—N1^i^	180.0	H2A—C2—H2B	107.3
N1^i^—Ni1—N2^i^	94.72 (19)	C1—C2—H2A	108.1
N1^i^—Ni1—N2	85.28 (19)	C1—C2—H2B	108.1
N1—Ni1—N2^i^	85.28 (19)	C3—C2—H2A	108.1
N1—Ni1—N2	94.72 (19)	C3—C2—H2B	108.1
N2—Ni1—O1^i^	86.26 (12)	C3—C2—C1	116.8 (6)
N2—Ni1—O1	93.74 (12)	C15—C14—H14	119.9
N2^i^—Ni1—O1^i^	93.74 (12)	C15—C14—C13	120.1 (6)
N2^i^—Ni1—O1	86.26 (12)	C13—C14—H14	119.9
N2—Ni1—N2^i^	180.0	C18—C19—C18^iii^	119.8 (6)
O2^ii^—Ni2—O2	180.0	C18^iii^—C19—C20	120.1 (3)
N3^ii^—Ni2—O2^ii^	89.28 (14)	C18—C19—C20	120.1 (3)
N3—Ni2—O2	89.28 (14)	H7A—C7—H7B	107.5
N3^ii^—Ni2—O2	90.72 (14)	C6—C7—H7A	108.4
N3—Ni2—O2^ii^	90.72 (14)	C6—C7—H7B	108.4
N3^ii^—Ni2—N3	180.0	C6—C7—C8	115.6 (5)
N4—Ni2—O2^ii^	91.61 (15)	C8—C7—H7A	108.4
N4^ii^—Ni2—O2	91.61 (15)	C8—C7—H7B	108.4
N4^ii^—Ni2—O2^ii^	88.39 (15)	N1—C1—C2	112.6 (5)
N4—Ni2—O2	88.39 (15)	N1—C1—H1A	109.1
N4—Ni2—N3^ii^	85.84 (19)	N1—C1—H1B	109.1
N4^ii^—Ni2—N3^ii^	94.16 (19)	C2—C1—H1A	109.1
N4^ii^—Ni2—N3	85.84 (19)	C2—C1—H1B	109.1
N4—Ni2—N3	94.16 (19)	H1A—C1—H1B	107.8
N4^ii^—Ni2—N4	180.00 (4)	C35—C34—C33	119.5 (5)
O3—S1—O2	110.02 (18)	C35—C34—H34	120.3
O3—S1—O1	108.6 (2)	C33—C34—H34	120.3
O3—S1—O4	109.5 (2)	C25—C26—H26A	109.8
O2—S1—O4	108.8 (2)	C25—C26—H26B	109.3
O1—S1—O2	109.74 (19)	C25—C26—H26C	109.4
O1—S1—O4	110.2 (2)	H26A—C26—H26B	109.5
S1—O2—Ni2	135.8 (2)	H26A—C26—H26C	109.5
Ni2—N3—H3	107.2	H26B—C26—H26C	109.5
C10—N3—Ni2	105.2 (3)	C27^iii^—C28—C29	120.2 (3)
C10—N3—H3	107.2	C27—C28—C29	120.2 (3)
C6—N3—Ni2	116.0 (3)	C27^iii^—C28—C27	119.7 (7)
C6—N3—H3	107.2	O8—C36—O7	123.0 (6)
C6—N3—C10	113.6 (5)	O8—C36—C33	124.7 (5)
S1—O1—Ni1	127.61 (19)	O7—C36—C33	112.3 (5)
Ni1—N1—H1	106.8	N3—C6—C7	111.9 (6)
C1—N1—Ni1	115.6 (3)	N3—C6—H6A	109.2
C1—N1—H1	106.8	N3—C6—H6B	109.2
C5—N1—Ni1	106.5 (4)	C7—C6—H6A	109.2
C5—N1—H1	106.8	C7—C6—H6B	109.2
C5—N1—C1	113.8 (5)	H6A—C6—H6B	107.9
Ni1—N2—H2	107.6	C25—C27—C30	120.5 (5)
C4—N2—Ni1	105.4 (4)	C28—C27—C30	118.9 (5)
C4—N2—H2	107.6	C28—C27—C25	120.5 (6)
C3—N2—Ni1	115.7 (3)	C21—C23—C24	119.9 (3)
C3—N2—H2	107.6	C21^iii^—C23—C24	119.9 (3)
C3—N2—C4	112.6 (4)	C21—C23—C21^iii^	120.2 (6)
Ni2—N4—H4	107.3	N2—C4—H4A	110.0
C9—N4—Ni2	105.7 (3)	N2—C4—H4B	110.0
C9—N4—H4	107.3	N2—C4—C5^i^	108.6 (4)
C8—N4—Ni2	115.8 (4)	H4A—C4—H4B	108.3
C8—N4—H4	107.3	C5^i^—C4—H4A	110.0
C8—N4—C9	113.1 (5)	C5^i^—C4—H4B	110.0
C35—C30—C31	116.6 (5)	C12—C13—C14	122.8 (5)
C35—C30—C27	119.2 (5)	C12—C13—H13	118.6
C31—C30—C27	124.2 (5)	C14—C13—H13	118.6
O6—C11—O5	123.1 (6)	C12—C17—H17	119.6
O6—C11—C12	124.2 (6)	C12—C17—C16	120.7 (6)
O5—C11—C12	112.8 (5)	C16—C17—H17	119.6
C21—C18—C15	117.5 (5)	C15—C16—C17	122.1 (5)
C19—C18—C15	122.3 (4)	C15—C16—H16	119.0
C19—C18—C21	120.1 (5)	C17—C16—H16	119.0
C14—C15—C18	123.7 (6)	N1—C5—C4^i^	109.2 (5)
C16—C15—C18	119.0 (5)	N1—C5—H5A	109.8
C16—C15—C14	117.3 (5)	N1—C5—H5B	109.8
C11—O5—H5	109.5	C4^i^—C5—H5A	109.8
C30—C35—H35	118.5	C4^i^—C5—H5B	109.8
C30—C35—C34	123.0 (5)	H5A—C5—H5B	108.3
C34—C35—H35	118.5	N2—C3—C2	112.1 (5)
C36—O7—H7	109.5	N2—C3—H3A	109.2
C25—C24—C25^iii^	121.4 (7)	N2—C3—H3B	109.2
C25^iii^—C24—C23	119.3 (4)	C2—C3—H3A	109.2
C25—C24—C23	119.3 (4)	C2—C3—H3B	109.2
N3—C10—H10A	110.0	H3A—C3—H3B	107.9
N3—C10—H10B	110.0	N4—C9—C10^ii^	108.2 (5)
N3—C10—C9^ii^	108.7 (5)	N4—C9—H9A	110.1
H10A—C10—H10B	108.3	N4—C9—H9B	110.1
C9^ii^—C10—H10A	110.0	C10^ii^—C9—H9A	110.1
C9^ii^—C10—H10B	110.0	C10^ii^—C9—H9B	110.1
C13—C12—C11	120.7 (5)	H9A—C9—H9B	108.4
C13—C12—C17	116.8 (5)	C21—C22—H22A	109.3
C17—C12—C11	122.5 (6)	C21—C22—H22B	109.8
C32—C33—C36	120.2 (5)	C21—C22—H22C	109.3
C34—C33—C32	118.5 (5)	H22A—C22—H22B	109.5
C34—C33—C36	121.2 (5)	H22A—C22—H22C	109.5
C33—C32—H32	119.4	H22B—C22—H22C	109.5
C31—C32—C33	121.1 (5)	N4—C8—C7	110.6 (5)
C31—C32—H32	119.4	N4—C8—H8A	109.5
H29A—C29—H29B	109.5	N4—C8—H8B	109.5
H29A—C29—H29C	109.5	C7—C8—H8A	109.5
H29B—C29—H29C	109.5	C7—C8—H8B	109.5
C28—C29—H29A	109.5	H8A—C8—H8B	108.1
C28—C29—H29B	109.5	C19—C20—H20A	109.5
C28—C29—H29C	109.5	C19—C20—H20B	109.5
C24—C25—C26	121.3 (5)	C19—C20—H20C	109.5
C24—C25—C27	118.9 (6)	H20A—C20—H20B	109.5
C27—C25—C26	119.7 (5)	H20A—C20—H20C	109.5
C30—C31—H31	119.4	H20B—C20—H20C	109.5
			
Ni1—N1—C1—C2	−54.6 (6)	C25^iii^—C24—C23—C21^iii^	102.8 (3)
Ni1—N1—C5—C4^i^	40.7 (4)	C25—C24—C23—C21	102.8 (3)
Ni1—N2—C4—C5^i^	−41.7 (5)	C25—C24—C23—C21^iii^	−77.2 (3)
Ni1—N2—C3—C2	56.4 (6)	C25^iii^—C24—C23—C21	−77.2 (3)
Ni2—N3—C10—C9^ii^	−42.8 (5)	C31—C30—C35—C34	−2.0 (8)
Ni2—N3—C6—C7	55.0 (5)	C31—C30—C27—C25	−80.6 (7)
Ni2—N4—C9—C10^ii^	41.2 (5)	C31—C30—C27—C28	103.0 (5)
Ni2—N4—C8—C7	−58.2 (5)	C21—C18—C15—C14	−107.6 (6)
O3—S1—O2—Ni2	−44.7 (3)	C21—C18—C15—C16	69.7 (7)
O3—S1—O1—Ni1	−75.3 (3)	C21—C18—C19—C18^iii^	−2.5 (3)
O2—S1—O1—Ni1	164.4 (2)	C21—C18—C19—C20	177.5 (3)
O1—S1—O2—Ni2	74.7 (3)	C14—C15—C16—C17	3.4 (9)
O4—S1—O2—Ni2	−164.6 (2)	C19—C18—C15—C14	76.6 (6)
O4—S1—O1—Ni1	44.6 (3)	C19—C18—C15—C16	−106.1 (6)
C30—C35—C34—C33	2.3 (8)	C19—C18—C21—C23	5.0 (7)
O6—C11—C12—C13	−5.3 (9)	C19—C18—C21—C22	−173.3 (4)
O6—C11—C12—C17	174.5 (6)	C1—N1—C5—C4^i^	169.2 (4)
C11—C12—C13—C14	−177.4 (5)	C1—C2—C3—N2	−70.0 (6)
C11—C12—C17—C16	178.1 (6)	C34—C33—C32—C31	0.2 (8)
C18—C15—C14—C13	174.6 (5)	C34—C33—C36—O8	170.1 (5)
C18—C15—C16—C17	−174.1 (5)	C34—C33—C36—O7	−9.0 (8)
C18—C21—C23—C24	177.6 (3)	C26—C25—C27—C30	6.9 (8)
C18—C21—C23—C21^iii^	−2.4 (3)	C26—C25—C27—C28	−176.8 (4)
C15—C18—C21—C23	−171.0 (4)	C36—C33—C32—C31	176.7 (5)
C15—C18—C21—C22	10.8 (7)	C36—C33—C34—C35	−177.8 (5)
C15—C18—C19—C18^iii^	173.3 (5)	C6—N3—C10—C9^ii^	−170.7 (4)
C15—C18—C19—C20	−6.7 (5)	C6—C7—C8—N4	73.2 (7)
C15—C14—C13—C12	−0.3 (9)	C27—C30—C35—C34	176.6 (5)
O5—C11—C12—C13	174.5 (6)	C27—C30—C31—C32	−177.7 (5)
O5—C11—C12—C17	−5.7 (8)	C27^iii^—C28—C27—C30	175.1 (5)
C35—C30—C31—C32	0.8 (8)	C27^iii^—C28—C27—C25	−1.3 (3)
C35—C30—C27—C25	101.0 (6)	C23—C24—C25—C26	−2.0 (5)
C35—C30—C27—C28	−75.5 (6)	C23—C24—C25—C27	178.7 (3)
C24—C25—C27—C30	−173.8 (4)	C4—N2—C3—C2	177.7 (5)
C24—C25—C27—C28	2.6 (7)	C13—C12—C17—C16	−2.1 (9)
C10—N3—C6—C7	177.0 (4)	C17—C12—C13—C14	2.8 (9)
C12—C17—C16—C15	−1.0 (10)	C16—C15—C14—C13	−2.8 (8)
C33—C32—C31—C30	0.1 (9)	C5—N1—C1—C2	−178.3 (5)
C32—C33—C34—C35	−1.3 (7)	C3—N2—C4—C5^i^	−168.6 (4)
C32—C33—C36—O8	−6.3 (9)	C3—C2—C1—N1	69.3 (6)
C32—C33—C36—O7	174.6 (5)	C9—N4—C8—C7	179.5 (4)
C29—C28—C27—C30	−4.9 (5)	C22—C21—C23—C24	−4.2 (5)
C29—C28—C27—C25	178.7 (3)	C22—C21—C23—C21^iii^	175.7 (5)
C25^iii^—C24—C25—C26	178.0 (5)	C8—N4—C9—C10^ii^	169.0 (5)
C25^iii^—C24—C25—C27	−1.3 (3)	C8—C7—C6—N3	−71.6 (7)

Symmetry codes: (i) −*x*+1/2, −*y*+3/2, −*z*+3/2; (ii) −*x*+3/2, −*y*+3/2, −*z*+3/2; (iii) −*x*+3/2, *y*, −*z*+1.

### catena-Poly[[[(1,4,8,11-tetraazacyclotetradecane-κ4N1,N4,N8,N11)nickel(II)]-µ2-sulfato-κ2O3:O4] hemi[4,4',4'',4'''-(2,2',4,4',6,6'-hexamethyl-[1,1'-biphenyl]-3,3',5,5'-tetrayl)tetrabenzoic acid] nonahydrate] (II) Hydrogen-bond geometry (Å, º)

**Table:**

*D*—H···*A*	*D*—H	H···*A*	*D*···*A*	*D*—H···*A*
N1—H1···O4	0.98	2.32	3.113 (5)	138
N2—H2···O3	0.98	2.42	3.267 (4)	144
N4—H4···O3^ii^	0.98	2.10	3.012 (5)	154
O5—H5···O4	0.82	1.79	2.597 (5)	169
O7—H7···O3^iv^	0.82	1.85	2.654 (5)	166

Symmetry codes: (ii) −*x*+3/2, −*y*+3/2, −*z*+3/2; (iv) *x*, −*y*+1/2, *z*−1/2.
